# Screening for latent tuberculosis in Norwegian health care workers: high frequency of discordant tuberculin skin test positive and interferon-gamma release assay negative results

**DOI:** 10.1186/1471-2458-13-353

**Published:** 2013-04-17

**Authors:** Gerd Gran, Jörg Aßmus, Anne Ma Dyrhol-Riise

**Affiliations:** 1 Department of Pulmonary Medicine, Haukeland University Hospital, Bergen, N-5021, Norway; 2 Centre for Clinical Research, Haukeland University Hospital, Bergen, N-5021, Norway; 3 Department of Clinical Science, University of Bergen, Bergen, N-5021, Norway; 4 Department of Internal Medicine, Section for Infectious Diseases, Haukeland University Hospital, Bergen, N-5021, Norway; 5 Present address: Department of Infectious Diseases, Oslo University Hospital, pb 4956 Nydalen, Oslo, 0424, Norway

**Keywords:** Tuberculosis, Quantiferon, Interferon-gamma release assay, IGRA, Screening, Health care workers, Low-endemic country, Norway

## Abstract

**Background:**

Tuberculosis (TB) presents globally a significant health problem and health care workers (HCW) are at increased risk of contracting TB infection. There is no diagnostic gold standard for latent TB infection (LTBI), but both blood based interferon-gamma release assays (IGRA) and the tuberculin skin test (TST) are used. According to the national guidelines, HCW who have been exposed for TB should be screened and offered preventive anti-TB chemotherapy, but the role of IGRA in HCW screening is still unclear.

**Methods:**

A total of 387 HCW working in clinical and laboratory departments in three major hospitals in the Western region of Norway with possible exposure to TB were included in a cross-sectional study. The HCW were asked for risk factors for TB and tested with TST and the QuantiFERON®TB Gold In-Tube test (QFT). A logistic regression model analyzed the associations between risk factors for TB and positive QFT or TST.

**Results:**

A total of 13 (3.4%) demonstrated a persistent positive QFT, whereas 214 (55.3%) had a positive TST (≥ 6 mm) and 53 (13.7%) a TST ≥ 15 mm. Only ten (4.7%) of the HCW with a positive TST were QFT positive. Origin from a TB-endemic country was the only risk factor associated with a positive QFT (OR 14.13, 95% CI 1.37 - 145.38, p = 0.026), whereas there was no significant association between risk factors for TB and TST ≥ 15 mm. The five HCW with an initial positive QFT that retested negative all had low interferon-gamma (IFN-γ) responses below 0.70 IU/ml when first tested.

**Conclusions:**

We demonstrate a low prevalence of LTBI in HCW working in hospitals with TB patients in our region. The “IGRA-only” seems like a desirable screening strategy despite its limitations in serial testing, due to the high numbers of discordant TST positive/IGRA negative results in HCW, probably caused by BCG vaccination or boosting due to repetitive TST testing. Thus, guidelines for TB screening in HCW should be updated in order to secure accurate diagnosis of LTBI and offer proper treatment and follow-up.

## Background

Tuberculosis (TB) presents globally a significant health problem and healthcare workers (HCW) are at increased risk of contracting latent TB infection (LTBI) and develop active TB [1,2]. The prevalence of TB infection and disease are highest among HCW in low/middle income countries [1]. The overall incidence of TB in Norway is 7/100.000 and of the approximately 350 new TB cases annually 80-90% are imported infections from TB-endemic countries [3]. According to the national guidelines those exposed for TB, including HCW, should be screened for TB infection and preventive anti-TB chemotherapy should be considered [4].

The tuberculin skin test (TST) has high sensitivity, but low specificity, especially in Bacillus Calmette-Guérin (BCG)-vaccinated individuals, because of cross-reactivity to non-tubercular mycobacterias (NTM) or due to booster-effect caused by repetitive testing [5]. This has resulted in long-term medical follow-up for many HCW with positive TST. There is no diagnostic gold standard test for LTBI, but blood based interferon-gamma release assays (IGRA) offer better specificity (98–100%) and at least as good sensitivity (70–97%) as the TST since they are unaffected by previous BCG vaccination and most NTM [2,6-9]. These assays, commercially available as QuantiFERON®TB Gold In-Tube (QFT) and T-SPOT.TB® measure *in-vitro* IFN-γ production by T-cells stimulated with the *M. tuberculosis*-specific antigens ESAT-6, CFP-10 and TB7.7. The tests have already been incorporated into national guidelines [10-13] and in Norway the QFT is recommended since 2007 as the test of choice following TST in a two-step approach [14]. Still, the role of IGRA in HCW screening is unclear [15].

Studies from TB low-endemic countries generally find a low prevalence of LTBI in HCW defined by IGRA [15,16,18-27]. A high proportion of discordant TST positive/IGRA negative results have been reported in HCW, especially in countries with high coverage of BCG vaccination [15,16,23,27-29]. In Norway a limited number of studies of the performance of IGRA in various populations have been published [23,30-33], but no study has focused on the routine screening of HCW. We have previously shown in a cohort with a predominance of immigrants that although one third tested QFT positive, in HCW, a subgroup of the study participants, only 10% had a positive QFT test [32].

We have performed a cross-sectional study amongst HCW in three major hospitals in the Western region of Norway to study the prevalence of QFT positivity in HCW working in hospital departments with possible risk of TB infection, identify possible risk factors for LTBI in our hospitals and investigate the performance of the QFT test in routine testing of HCW.

## Methods

### Study participants

HCW working in out-patient TB clinics and wards at the departments of Pulmonary medicine and Infectious medicine or in the laboratories at the departments of Microbiology and Pathology in three major hospitals in the Western region of Norway were included in the study in the period from September 2008 to September 2009. All HCW had possible contacts with patients with diagnosed or suspected infectious TB, infected biological material or TB cultures as part of clinical work or diagnostic procedures and consisted of medical doctors, nurses, laboratory staff and administrative personnel. The study participants with a positive QFT were followed according to clinical practice with clinical and radiological examination to rule out active TB. Treatment of LTBI for three months was offered in accordance with the Norwegian recommendations [4]. The following variables were registered using a standardized questionnaire: age, sex, workplace, occupation, country of origin, year of immigration, previous TB, stay (>2 weeks) or work in a TB endemic country, occupational exposure to TB in the actual hospital or outside the hospital (included working in hospitals in TB-endemic countries), prior TST and BCG vaccination, also assessed through inspection for scars. A TST (0.1 ml tuberculin PPD RT23 2 TU, SSI, Copenhagen, DK) was performed after blood was collected for QFT to avoid the possibility of boosting and read after 72 hours [4]. In some subjects no TST was performed due to earlier strong reactions or previous TB infection [34].

Inclusion in the study was voluntary and written informed consent was given from all the study participants before inclusion in the study. The study was approved by the Regional Ethics Committee for Medical Research (REK-vest) and the Norwegian Data Inspectorate.

### QuantiFERON TB-gold in-tube assay

Blood was drawn from all HCW at their respective hospitals and analysed with the QuantiFERON® TB-Gold In-tube assay (QFT), (Cellestis/Qiagen) at the laboratory for Infectious diseases at Haukeland University hospital. All HCW with a positive or inconclusive QFT test were offered retesting. The HCW with negative first test were not retested. The blood samples were treated as recommended by the manufacturer. Briefly, one ml of whole blood was sampled in each of the three QFT tubes containing either TB specific antigen (ESAT-6, CFP-10 and TB7.7), no antigen (negative control) or mitogen antigen (positive control) and the IFN-γ concentrations (IU/ml) in plasma was measured by an ELISA reader and calculated by the ‘QFT-TB-analysis Software’. An IFN-γ ≥ 0.35 IU/ml (TB antigens minus negative control) was considered a positive test.

### Statistics

Statistical analysis was performed using SPSS 20 and the graphics was done by Matlab 2010a. Data are presented as median values with range. Univariate assessment of risk factors for positive QFT and TST ≥ 15 mm was done by Chi square test and *t*-test as appropriate.

The associations between QFT and TST as dependent variables and age, sex, country of origin, stay in TB endemic country and TB exposure as predictors were analyzed by a logistic regression model. We considered a fully adjusted multivariate model containing all predictors as well as an univariate model for each predictor. We selected 15 mm as cut-off to dichotomize TST. The general significance level was set to 0.05.

## Results

### Study population

A total of 387 HCW working in the clinical departments diagnosing and treating TB patients or in the laboratories handling possible TB infected biological samples were included in the study (Table 1). The majority of the study participants were female (84.2%) and the median age was 36. Most of them were ethnically Norwegians (92.2%) and only 15 (3.9%) had migrated from TB high-endemic regions, predominately from Asia. The majority of the HCW were nurses (57.4%), 15.5% were physicians, 25.6% were laboratory staff and 1.6% were administration staff. A history of BCG vaccination was reported in 97.9% and the time since BCG-vaccination was median 21 years. Most of the foreign-born were vaccinated as infants or young children, whereas the Norwegians were vaccinated at the age of fourteen according to the Norwegian national vaccination program [4]. Three HCW (0.8%), all from TB low-endemic countries, had previously been treated for active TB disease (8, 13 and 29 years back). A total of 74.4% of the HCW reported stay in a TB-endemic country, most for shorter time-periods and only 34% stayed for a minimum of three months, whereas 59 (15.2%) HCW had been working in a TB-endemic country, but only 21 of them worked for a period of three months or more. The median time since the last work period was five years (range 0-33). A total of 61.8% of the HCW reported known exposure to an infectious TB patient at their workplace and 22% reported contact with TB infected biological material. The majority of the HCW reported use of infection control equipment during contact with TB patients or infectious material (82.4% and 83.2%, respectively). Known exposure to TB outside work, including at foreign hospitals, was reported in 50 (12.9%) HCW.

**Table 1:** Characteristics of study participants (n = 387)

**Characteristics**	**No**	**(%)**
Age ^1)^	36	23-64
Sex		
*Male*	61	15.8
*Female*	326	84.2
Origin		
*Norway*	357	92.2
*TB low-endemic country*	15	3.9
*TB high-endemic country*	15	3.9
Work place		
*Infectious Medicine*	160	41.3
*Pulmonary Medicine*	123	31.8
*Microbiology*	80	20.7
*Pathology*	24	6.2
Profession		
*Nurse*	222	57.4
*Physician*	60	15.5
*Laboratory staff*	99	25.6
*Administration*	6	1.6
Work TB endemic country ^2)^	59	15.2
Stay TB endemic country ^2)^	288	74.4
Exposure to TB at work ^3)^		
*Patients*	239	61.8
*Laboratory*	85	22.0
Exposure to TB outside work	50	12.9
BCG vaccinated	379	97.9
BCG scar	358	92.5
Previous TB	3	0.8

^1)^Median (range). Definitions: TB = Tuberculosis. ^2)^The three longest visits were registered. ^3)^The three longest contacts were registered.

### Characteristics of health care workers with a positive QuantiFERON-TB test

A positive QFT result was observed in 18 HCW when first tested (median 0.76 IU/ml, range 0.39-7.9 IU/ml) (Table 2). These HCW, except two refusing to repeat the test, were retested twice or three times. Thus, after retesting 13 (3.4%) of all the HCW had a persistent positive QFT, indicating LTBI. Two HCW tested positive the first time (0.40-0.76 IU/ml), then negative the second test (0.30 IU/ml), whereas both reverted to positive (0.49-0.93 IU/ml) when tested for the third time and were concluded as positive. The additional five HCW with a positive initial QFT that retested negative all had low initial responses (0.39-0.66 IU/ml). In two other HCW the QFT was initially indeterminate, but negative when retested.

**Table 2:** Characteristics of HCW with positive or indeterminate QuantiFERON-TB

**HCW**	**Age**	**Sex**	**1. QFT (IU/ml)**	**Origin**	**Occupation**	**BCG**	**TST ≥ 6**	**2. QFT (IU/ml)**
1	22	F	4.51	Norway	Laboratory	no	yes	9.51
2	35	F	0.57	Norway	Laboratory	yes	yes	−0.05
3	26	F	0.66	Norway	Laboratory	yes	yes	−0.01
4	41	F	0.40	Asia	Laboratory	yes	yes	0.49^1^
5	34	M	0.76	Norway	Laboratory	yes	no	0.93^1^
6	32	F	IND	Norway	Laboratory	yes	yes	0.05
7	24	F	1.60	Norway	Nurse	yes	yes	1.09
8	59	F	0.47	Norway	Nurse	yes	^2^	0.49
9	39	F	0.39	Norway	Secretary	yes	yes	−0.01
10	44	F	0.40	W. Europe	Physician	yes	yes	^2^
11	46	M	1.09	Norway	Physician	yes	yes	1.36
12	29	M	0.46	Norway	Physician	yes	no	−0.14
13	54	M	2.31	Norway	Physician	yes	yes	2.66
14	44	M	1.66	N. America	Physician	no	yes	^2^
15	28	M	IND	Norway	Nurse	yes	yes	neg
16	25	F	7.90	Africa	Nurse	yes	no	pos^3^
17	60	F	5.85	Asia	Nurse	yes	yes	4.15
18	60	F	0.68	Norway	Assistant	yes	yes	neg^3^
19	65	F	1.31	Norway	Nurse	yes	yes	2.17
20	33	M	5.51	Norway	Physician	yes	yes	>10

^1^Third QFT, second test 0.30 UI/ml in both HCW 4 and 5. ^2^No test was performed. ^3^No value was given.

QFT = QuantiFERON-TB. IND = indeterminate.

The characteristics of the 13 HCW with a persistent positive QFT were as follows; the HCW worked in different hospitals in different departments. Five were nurses, five medical doctors and three laboratory staff. One HCW had been diagnosed with active TB 29 years back, but had only received treatment for four months. It is of note that the two others with previous active TB were QFT negative. Ten QFT positive HCW were born in western TB low-endemic countries, the majority in Norway and three had origin from TB-endemic regions. Eleven of the QFT positive HCW were BCG vaccinated with visible scars. Eight HCW reported stay, but only two reported working in a TB-endemic region. All QFT positive HCW reported known exposure to TB during work at the hospital in Norway. Ten HCW reported contact with an infectious TB patient, eight HCW at two or more occasions and six had performed or assisted during bronchoscopy. Nine of these had used recommended infection control equipment at regular basis. Three HCW had been in contact with TB infected biological material during diagnostic handling or procedures and had for most of the occasions used infection control equipment. Seven HCW had also been in contact with TB-patients outside the hospital. In the univariate logistic regression analysis female and country of origin were significant associated with a positive QFT, whereas in the multivariate analysis only country of origin remained significant (Table 3). Clinical and radiological examination did not show any signs of active TB in any of the HCW. They were offered preventive TB therapy after medical evaluation, but only one was treated.

**Table 3:** Multivariate analysis of risk factors associated with a positive QFT

	**Univariate (N = 13)**		**Multivariate (N = 13)**	
**Characteristics**	**OR (95% CI)**	**P-value**	**OR (95% CI)**	**P-value**
Female	3.54 (1.12, 11.21)	.031	2.59 (0.76, 8.86)	.131
Age	1.04 (0.99, 1.09)	.141	1.03 (0.98, 1.08)	.259
Origin	9.05 (2.20, 37.16)	.002	**14.13 (1.37, 145.38)**	**.026**
Stay in TB endemic country	0.74 (0.22, 2.45)	.681	0.80 (0.31, 2.04)	.632

OR; odds ratio; CI; confidence interval; OR per standard-deviation increase for continuous variables Age; Origin; country of birth, divided into three groups, 1) Norway, 2) other TB low- endemic countries, 3) TB endemic countries; Stay in TB endemic country; stayed and/or worked in TB endemic country (>2 weeks).

Exposure to TB was too unbalanced to produce definite confidence intervals and therefore not reported.

### Discordant tuberculin skin tests and QuantiFERON-TB results in health care workers

A total of 214 (55.3%) demonstrated a positive TST (≥ 6 mm) and 13.7% had even TST ≥ 15 mm (Table 4). In 19 (4.9%) a TST was not performed due to earlier strong reactions or previous active TB. In the multivariate analysis there was no significant association between risk factors for TB infection and TST ≥ 15 mm (Table 5).

**Table 4:** QuantiFERON-TB results in HCW for various TST reactions

**Induration (mm)**	**TST (n = 387)**	**QFT (n = 13)**
	**n (%)**	**n (% of TST group)**
≥ 6	214 (55.3)	10 (4.7)
0-5	154 (39.8)	2 (1.3)
6-10	85 (22.0)	4 (4.7)
11-14	76 (19.6)	4 (5.3)
≥ 15	53 (13.7)	2 (3.8)
No TST^1^	19 (4.9)	1 (5.3)

TST = tuberculin skin test. QFT = QuantiFERON-TB.

^1)^Not tested due to previous strong response or previous active TB.

**Table 5:** Multivariate analysis of risk factors associated with a TST ≥ 15 mm

	**Univariate (n = 384)**		**Multivariate (n = 53)**	
**Characteristics**	**OR (95% CI)**	**P-value**	**OR (95% CI)**	**P-value**
Female	0.34 (0.08, 1.47)	.148	0.27 (0.06, 1.21)	.088
Age	1.01 (0.98, 1.05)	.413	1.02 (0.98, 1.05)	.382
Origin	1.66 (0.36, 7.69)	.519	1.16 (0.19, 7.16)	.871
Stay in TB endemic country	0.86 (0.38, 1.92)	.705	0.42 (0.12, 1.55)	.193
Exposure to TB	0.79 (0.34, 1.84)	.588	0.79 (0.33, 1.90)	.598

OR; odds ratio; CI; confidence interval; OR per standard-deviation increase for continuous variables Age; Origin; country of birth, divided into three groups, 1) Norway, 2) other TB low- endemic countries, 3) TB endemic countries; Stay in TB endemic country; stayed and/or worked in TB endemic country (>2 weeks); Exposure to TB; exposed to TB at work, either in direct contact with patients or in a laboratory.

**Figure 1:**
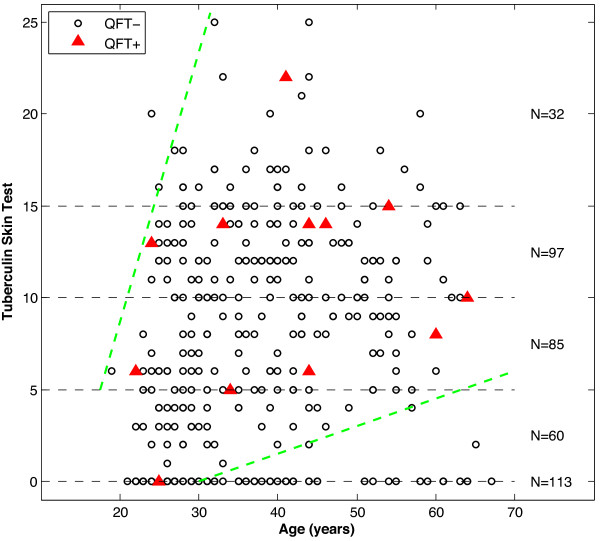
**Relationship between age, tuberculin skin test and QFT.** The red triangles show the study participants with a positive QFT (n = 12). The circles show the study participants with a negative QFT (n = 355). One QFT positive was not included since no TST was performed.

Only 4.7% of the HCW with a positive TST ≥ 6 mm were QFT positive and there was a wide range of TST reactions seen in the QFT positive group (Table 4). Only two of the 53 (3.8%) HCW with TST ≥ 15 mm demonstrated a positive QFT and none one of the two HCW with previously strong TST responses > 25 mm were QFT positive. In contrast, two of the TST negative HCW (1.3%) were QFT positive, one with origin from a TB endemic country and the other had been exposed for TB at work in the laboratory (Table 2; HCW 5 and 16, respectively). The association between TST and QFT for the various age groups is shown in Figure 1.

## Discussion

We present a cross sectional study on routine IGRA testing of HCW working in three major hospitals in the Western region of Norway, a TB low-endemic country. We find that only 3.4% of the HCW tested positive with QFT in contrast to 55.3% with TST, indicating low level of LTBI in HCW working at Norwegian hospitals. This is in support of a previous Norwegian contact investigation study that also found a frequency of 3% of LTBI among HCW by the T-SPOT.TB test [23] and a Danish study from hospital medical wards where only 1% of the HCW had a positive QFT [16]. In contrast, in a study from Russia, a TB high-endemic country, 47% of the HCW in the TB department were IGRA positive [17]. We did not find any effect of age on the QFT results. In the study by Nienhaus *et al*, however, IGRA results among HCW depended greatly on age and country with a QFT positivity of 25% in Germany, 45% in Portugal and 33% in France in the age group over 55 years [26]. In a recent review from Zwerling *et al*. the pooled prevalence of positive IGRA using either test was significantly lower than for a positive TST among HCW in low TB incidence settings, and IGRA showed good correlation with occupational risk factors for TB exposure [29].

The 13 HCW with a persistent positive QFT were equally distributed between the three hospitals and between nurses, medical doctors and laboratory staff and the majority was born in Norway. All HCW reported known exposure to TB at work, but had used infection control equipment during patient care and diagnostic procedures. Seven HCW reported additional possible environmental exposure to TB. Further, four of the HCW had either origin from and/or reported working in TB high-endemic regions. It is likely that these were infected abroad since country of origin was the only risk factor significantly associated with a positive QFT test in the multivariate analysis. One HCW had been diagnosed with active TB several years back illustrating that IGRAs could stay positive long after treated infection [32]. From 2005 a total of 47 HCW with TB infection, of whom 14 developed active TB, have been reported in Norway (personal communication, The Norwegian Institute of Public Health). However, 25 were born outside Norway possibly implying that TB exposure had occurred abroad. Based on our results we cannot conclude for sure, but altogether this indicates a low risk of contracting TB and a good infectious control at Norwegian hospitals.

The growing literature raises the question of whether IGRA could replace the TST when screening HCW for TB in routine practice or contact investigations due to the improved specificity [15,18,26-28]. This is especially relevant in countries like Norway with high coverage of BCG vaccination and discordant TST positive/IGRA negative results. In a Canadian study LTBI prevalence among HCW measured by the TST was low and the most common discordant test results were TST negative/QFT positive [35]. In contrast, we observed that a total of 33% of the HCW had TST > 10 mm, 13.7% had TST ≥ 15 mm with a frequency of only 4% QFT positivity and even HCW with the highest TST values > 25 mm were QFT negative. This might represent false negative QFT, but there was no association between known TB risk factors and TST ≥ 15 mm. BCG vaccination at the age of fourteen was performed according to the Norwegian national vaccination program from 1947 to 2009 and explains why 98% of all the HCW in our cohort were vaccinated. Thus, the discrepancy it most likely explained by false positive TST due to high BCG vaccination coverage or booster effects after repeatedly TST testing in the hospitals. With over half of the HCW in our study demonstrating a positive TST, which we believe is representative for HCW working at Norwegian hospitals, it is obvious the TST in our setting generally is of little use. Instead, IGRA offer better specificity and thereby lower prevalence of positive tests and fewer HCW who require X-rays, further clinical follow-up or LTBI treatment. Still, medical follow-up of HCW with strong TST reactions and risk factors for TB reactivation might be warranted since there is no diagnostic gold standard for LTBI.

There is concern about IGRA reproducibility, defining the optimum cut-off values for positive tests which most accurately distinguish new TB infection from random variation as well as defining the right interpretation of discordant TST and IGRA in serial screening [29,36,37]. In our study, the HCW with initial positive QFT that retested negative all had low initial IFN-γ responses below 0.70 IU/ml. Poor reproducibility of the assay is a more likely explanation than true reversions since the tests were performed within short periods. Such fluctuations in IGRA findings were also demonstrated in a study where HCW in contact with TB patients were tested monthly [38]. When an increase in QFT cut-off from < 0.35 to ≥ 0.70 IU/ml was applied inconsistent IGRA results were reduced from 52% to 27% and consistency in QFT results was associated with baseline IFN-γ levels. A recent review also concludes that subjects with baseline results around the diagnostic threshold are more likely to show inconsistent results on retesting [36]. Also in our cohort there was a tendency to higher IFN-γ levels in those with consistent positive QFT. Thus, a borderline zone from 0.20-0.70 IU/ml has been suggested in the routine screening of HCW with retesting before preventive chemotherapy is recommended [36]. Longitudinal research is therefore requested to inform guidelines on IGRA serial testing.

Some countries such as the USA have almost since their introduction recommended the two new IGRAs for screening of LTBI [11]. Others, including the Norwegian guidelines, have in general been more cautious and recommended a two-step approach with an initial TST [14]. The European guidelines conclude that IGRA may be used as part of the overall risk assessment to diagnose LTBI and identify individuals for preventive treatment [10]. In the updated guidelines from the USA the advantages and disadvantages of the various tests are discussed [39]. When choosing test one must consider the accuracy of the test in the specific risk groups as well as populations. The guidelines also conclude that due to the high negative predictive value, progression to active TB in healthy immune competent individuals with negative IGRA is very unlikely.

According to Norwegian guidelines HCW who has worked or stayed in a TB endemic country or are included in contact investigations should be screened for TB infection in a two-step approach with an initial TST before IGRA [14]. Many hospitals have also performed regular routine TST in HCW with risk for TB infection at their workplace. Our study demonstrating a high frequency of discordant TST positive/IGRA negative results indicates that the National guidelines should be updated and an “IGRA-only strategy” considered for HCW. Use of IGRA in this population is expected to increase diagnostic specificity and improve acceptance of treatment for LTBI. The higher per-test cost of IGRAs may be compensated for by lower post-screening costs (medical follow-up, chest x-rays and chemoprevention). A recent review of cost-effectiveness of IGRA concludes that in four studies, the “two-step strategy” and in two studies the “IGRA-only strategy” was most cost-effective [40]. A recent British study concludes that IGRA can be an institutional cost saving and result in higher compliance rates [41]. There is growing evidence in support of the use of IGRA in screening risk groups such as HCW. However, one must be aware of the uncertainty of IGRA during serial-testing with yet undefined proper cut-off levels and possible low-level false-positive IGRA results that will lead to unnecessary follow-up of low-risk HCW.

The limitations of our study are the small number of IGRA positive to draw strong conclusions on the overall prevalence of TB infection in HWC in Norway, the risk factors for TB infection and the effects of serial testing. In addition it is not possible to show the frequency of converters since no repetitive testing of the QFT negative were performed.

## Conclusions

We find in this cross sectional study that only 3.4% of HCW were QFT positive in contrast to 55.3% TST positive indicating a low level of LTBI among HCW working at hospitals in Western Norway. The only risk factor associated with a positive QFT was origin from a TB-endemic country implying a low risk for contracting TB infection at Norwegian hospitals. The “IGRA-only” seems like a desirable strategy, despite its limitations, due to the high number of false positive TST probably caused by BCG vaccination and repetitive TST testing. Thus, there is a need for an update of Norwegian guidelines for TB screening in HCW in order to get more accurate diagnosis of LTBI and offer proper treatment and follow-up.

## Competing interests

The authors declare that they have no competing interests.

## Authors’ contributions

GG: has participated in design of the study, recruiting study participants, interpretation of data, statistical analysis and drafted the manuscript. JA: has performed statistical analysis and revising of the manuscript. AMDR: has designed the study, participated in interpretation of data, statistical analysis and drafted the manuscript. All authors have read and approved the final manuscript.

## Pre-publication history

The pre-publication history for this paper can be accessed here:

http://www.biomedcentral.com/1471-2458/13/353/prepub
